# Construction of a Nomogram Based on a Hypoxia-Related lncRNA Signature to Improve the Prediction of Gastric Cancer Prognosis

**DOI:** 10.3389/fgene.2020.570325

**Published:** 2020-10-20

**Authors:** Qian Chen, Lang Hu, Kaihua Chen

**Affiliations:** ^1^Department of Research, Guangxi Medical University Cancer Hospital, Nanning, China; ^2^Department of Radiation Oncology, Guangxi Medical University Cancer Hospital, Nanning, China

**Keywords:** gastric cancer, lncRNA, TCGA, hypoxia-related prognostic signature, nomogram

## Abstract

**Background:**

Gastric cancer is one of the most common malignant tumors and has a poor prognosis. Hypoxia is related to the poor prognosis of cancer patients. We searched for hypoxia-related long non-coding RNAs (lncRNAs) to predict both overall survival (OS) and disease-free survival (DFS) of gastric cancer patients.

**Methods:**

We obtained hypoxia-related lncRNA expression profiles and clinical follow-up data of patients with gastric cancer from The Cancer Genome Atlas and the Molecular Signatures Database. The patients were randomly divided into a training group, test group and combined group. The hypoxia-related prognostic signature was constructed by Lasso regression and Cox regression models, the prognoses in different groups were compared by Kaplan–Meier (K-M) analysis, and the accuracy of the prognostic model was assessed by receiver operating characteristic (ROC) analysis.

**Results:**

A hypoxia-related prognostic signature comprising 10 lncRNAs was constructed to predict both OS and DFS in gastric cancer. In the training, test and combined groups, patients were divided into high- and low-risk groups according to the formula. Kaplan–Meier analysis showed that patients in the high-risk group have poor prognoses, and the difference was significant in the subgroup analyses. Receiver operating characteristic analysis revealed that the predictive power of the model prediction is more accurate than that of standard benchmarks. The signature differed across *Helicobacter pylori* (Hp) status and T stages. Multivariate Cox analysis showed that the signature is an independent risk factor for both OS and DFS. A clinically predictive nomogram combining the lncRNA signature and clinical features was constructed; the nomogram accurately predicted both OS and DFS and had high clinical application value. Weighted correlation network analysis combined with enrichment analysis showed that the primary pathways were the PI3K-Akt, JAK-STAT, and IL-17 signaling pathways. The target genes NOX4, COL8A1, and CHST1 were associated with poor prognosis in the Gene Expression Profiling Interactive Analysis, Gene Expression Omnibus, and K-M Plotter databases.

**Conclusions:**

Our 10-lncRNA prognostic signature and nomogram are accurate, reliable tools for predicting both OS and DFS in gastric cancer.

## Introduction

Gastric cancer is one of the most serious types of malignancies worldwide, ranking fifth in the number of new cases and third in the number of tumor-related deaths. In 2018, a total of 1,033,701 new cases and 782,685 deaths occurred, resulting in a massive social burden (Bray et al., 2018). In West Asian countries, especially Iran, Turkmenistan, and Kyrgyzstan, gastric cancer has a high mortality rate. Despite recent advances in surgical treatment, radiotherapy, and chemotherapy for gastric cancer, the overall survival (OS) rate of gastric cancer, especially advanced gastric cancer, is still very low. The tumor node metastasis (TNM) staging system is currently the gold standard for evaluating cancer prognosis, but treatment responses and prognoses of patients in the same stage differ in practice because of uncharacterized genetic alterations (Choi et al., 2017). Therefore, new markers are needed to predict the prognosis of patients with gastric cancer.

Long non-coding RNAs (lncRNAs) are non-protein-coding RNAs at least 200 nucleotides in length (Ponting et al., 2009). With the development of high-throughput sequencing technology, an increasing number of lncRNAs have been reported to affect the occurrence and development of tumors (Martens-Uzunova et al., 2014; Bhan et al., 2017). Long non-coding RNAs have also been reported to be prognostic biomarkers for many cancers, including gastric cancer, lung cancer, pancreatic cancer, hepatocellular carcinoma, and colorectal cancer (Peng and Jiang, 2016; Zhao et al., 2017; Xie et al., 2018; Huang et al., 2019; Wang and Qin, 2019). Recently, lncRNA-based signatures have attracted much attention due to their high predictive accuracy (Miao et al., 2019; Liu et al., 2020; Shen et al., 2020). Hypoxia is a main feature of the tumor microenvironment and is related to poor prognosis. Hypoxia promotes tumor cell proliferation and invasion, angiogenesis, treatment resistance and metastasis (Harris, 2002; Lu and Kang, 2010). Under hypoxic conditions, the lncRNA NORAD has been reported to promote pancreatic cancer metastasis via epithelial-mesenchymal transition (EMT; Li et al., 2017). In oral squamous cell carcinoma, the expression of the lncRNA HAS2-AS1 is upregulated in hypoxic tumor tissues, which promotes tumor invasiveness (Zhu et al., 2017). However, prognostic markers based on hypoxia-related lncRNA expression profiles have not been studied in gastric cancer.

The purpose of this article is to construct a hypoxia-related lncRNA signature and nomogram with a bioinformatics approach in order to improve the ability to predict both OS and disease-free survival (DFS) of gastric cancer patients. First, we obtained the expression profiles and clinical data of gastric cancer patients from The Cancer Genome Atlas (TCGA) database and extracted multiple hypoxia-related lncRNAs from the Molecular Signatures Database (MSigDB). Then, Lasso regression and Cox regression models were used to construct a hypoxia-related prognostic signature to predict both OS and DFS of gastric cancer patients and to verify the accuracy of the model in different data sets. Finally, we constructed a prognostic nomogram based on the hypoxia-related lncRNA prognostic signature and clinical characteristics. Both the predictive accuracy and clinical application value of our nomogram were higher than those of the TNM staging system, offering new tools for the prognostic prediction and treatment of gastric cancer.

## Materials and Methods

### Data Collection

We downloaded FPKM RNA-seq data and clinical data for patients with stomach adenocarcinoma (STAD) from the TCGA database^1^; the dataset contained a total of 375 gastric cancer samples and 32 tumor-adjacent samples. The clinical data included age, sex, stage, grade, *Helicobacter pylori* (Hp) status, survival time, and outcome. The exclusion criteria were set as follows: (1) histologic diagnosis is not STAD; (2) samples without completed data for analysis; and (3) clinical follow-up time of less than 30 days. Overall, a total of 334 STAD patients from the TCGA database were included in our study. The STAD RNA-seq data were reannotated according to the ENSEMBL database^2^ and separated into lncRNAs and mRNAs. Gene expression data and corresponding clinical information of gastric cancer patients were obtained from the Gene Expression Omnibus (GEO) database.^3^ An independent dataset (GSE84426) was included in the study, which including expression profiles and clinical information for 76 patients with gastric cancer. The gene expression data were generated using the Illumina HumanHT-12 V3.0 expression BeadChip platform. When multiple probes mapped to the same gene, we used median values to represent the expression of that gene. We deleted the probe when one probe correspond to multiple genes. Rows of RNA data with no expression or a mean count of <0.5 were deleted. Batch normalization using the “sva” and “limma” package in R.

### Identification of Hypoxia-Related lncRNAs and Differentially Expressed Genes

The hypoxia-related gene set was downloaded from MSigDB (Genes known to be induced by hypoxia M10508; Cellular response to hypoxia, M26925),^4^ and a total of 151 hypoxia-related genes were obtained. These genes were used to establish a hypoxia score of gastric cancer genes by gene set enrichment analysis (GSEA). The correlation between the hypoxia score and the expression of lncRNAs in gastric cancer patients was analyzed by coexpression analysis. A total of 436 hypoxia-related lncRNAs were identified (| cor| > 0.4, *P* value < 0.05). In addition, we identified differentially expressed genes using the “limma” package in R. The cutoff values were determined based on the following parameters: | logFC| > 1 and FDR < 0.05.

### Identification of a Hypoxia-Related lncRNA Prognostic Signature

In the training group, univariate Cox regression analysis was used to identify prognosis-related lncRNAs (*P* < 0.05). A lncRNA signature to predict the prognosis of gastric cancer patients was constructed by Cox regression with the Lasso method using the “glmnet” package in R. The lambda.1se, a penalty parameter used to prevent overfitting effects of the model, was selected using 1000 times ten-fold cross validation. We used the following formula to calculate the risk score of gastric cancer patients: Risk Score = ΣβlncRNAi × ExplncRNAi (where β is the coefficient and Exp is the expression level of the lncRNA). The patients were divided into high- and low-risk groups according to the median score, and the differences in OS and DFS were compared. In addition, the same formula was used to calculate the risk score in the test group and the combined group to verify the accuracy of the model.

### Construction and Evaluation of the Nomogram

We used the R packages “Hmisc,” “lattice,” “Formula,” “foreign,” and “rms” to construct nomograms to predict OS and DFS of gastric cancer patients. A calibration plot was established to evaluate the predictive accuracy of the nomogram. Decision curve analysis was used to assess the net benefit of the predicted nomogram to patients during clinical decision making. The abscissa of the curve shows the potential probability threshold; the ordinate, the net benefit.

### Weighted Correlation Network Analysis and Functional Enrichment Analysis

Using weighted correlation network analysis (WGCNA) with the R package “WGCNA,” we constructed a coexpression network from the gene model and incorporated clinical characteristics (including the risk score of the hypoxia-related lncRNA prognostic signature). The soft thresholding power was selected as 2 for construction of the weighted network. The included genes were divided into six modules. The most statistically significant module considering the risk score was selected, and the genes in this module were used to analyze the potential biological mechanisms of the hypoxia-related lncRNAs in the prognostic signature. Then, GO and KEGG analyses were performed with the R package “clusterProfiler” to analyze the functions and pathways enriched with the target genes. The pathway analysis data set (c2.cp.kegg.v7.0.symbols) downloaded from MSigDB and GSEA 4.0.3 was used to analyze the differences in the activity and expression patterns of the pathways and their constituents between the high-risk group and the low-risk group. A two-sided *P* value of <0.05 was considered significant in the enrichment analysis.

### Construction of a Protein–Protein Interaction Network and Verification of Target Genes

We used the STRING online database to analyze Protein–Protein Interactions (PPIs) and Cytoscape software to visualize PPI networks. The Gene Expression Profiling Interactive Analysis (GEPIA) database^5^ contains sequencing and follow-up data compiled from the TCGA and GTEx after standardized processing; specifically, dataset GSE84426 contains the gene expression and follow-up data of 76 gastric cancer patients. The Kaplan–Meier (K-M) Plotter database^6^ includes gene chip expression profiles and prognostic data for 875 cases of gastric cancer. The above three databases were used to verify the relationship between the target genes and patient prognosis.

### Immune Infiltration Analysis

Data from gastric cancer patients relating to immune cell infiltration (B cells, CD4+ T cells, CD8+ T cells, neutrophils, macrophages, and dendritic cells) were downloaded from the Tumor Immune Estimation Resource (TIMER) database.^7^ Spearman’s test was used to measure correlations between genes and the immune microenvironment. A two-tailed *P* value of <0.05 was considered statistically significant.

### Data Analysis

The chi-square test and Fisher’s exact probability test were used to analyze the relationships between clinical characteristics and the risk score. Kaplan–Meier analysis with the log-rank test was performed to analyze differences in OS and DFS between the high- and low-risk groups. Univariate and multivariate Cox analyses were used to compare the impact of the risk score on OS and DFS. The proportional hazards assumption was checked using the Schoenfeld residuals test and all variables showed *P* value >0.05, which fulfilled the assumption. The area under the curve (AUC) was implemented to evaluate the accuracy of the hypoxia-related lncRNA prognostic signature for predicting prognosis prediction, while receiver operating characteristic (ROC) analysis was used to determine the sensitivity and specificity. All data analysis was performed in R software (version number: 3.6.2), and a *P* value of <0.05 was considered statistically significant.

## Results

### Identification of Hypoxia-Related lncRNAs

**FIGURE 1:**
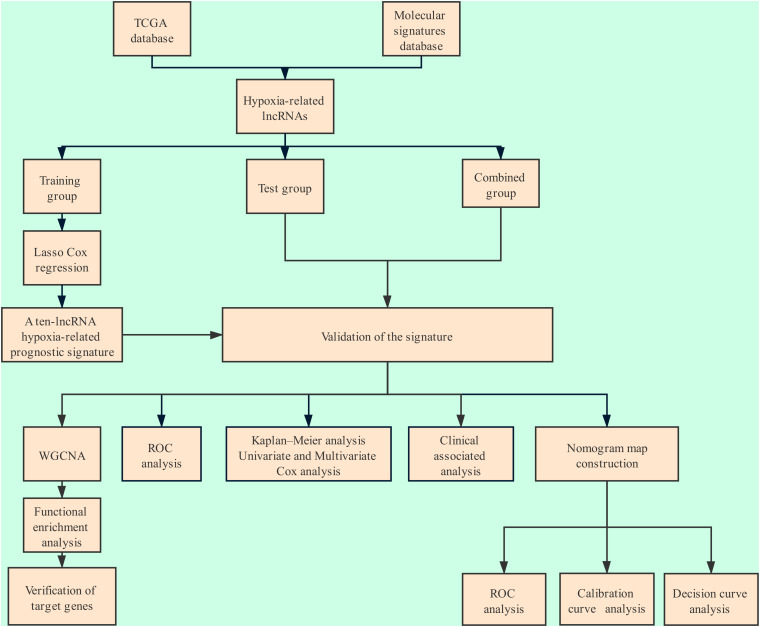
Flowchart of the analysis.

The flow chart of the entire study is shown in Figure 1. Samples from patients with a clinical follow-up of less than 30 days were excluded from the study. A total of 334 gastric cancer samples were included in the combined group, whick were randomly divided at into a training group (236 samples) and a test group (98 samples) at a ratio of 7:3. A total of 151 hypoxia-related genes (M10508 and M26925) were obtained from MSigDB. Through coexpression analysis, we identified 436 hypoxia-related lncRNAs (| cor| > 0.4, *P* value < 0.05). After performing univariate Cox analysis on the training group, we identified 24 candidate lncRNAs (*P* < 0.05) closely related to OS.

### Construction and Verification of the Hypoxia-Related lncRNA Prognostic Signature

**FIGURE 2:**
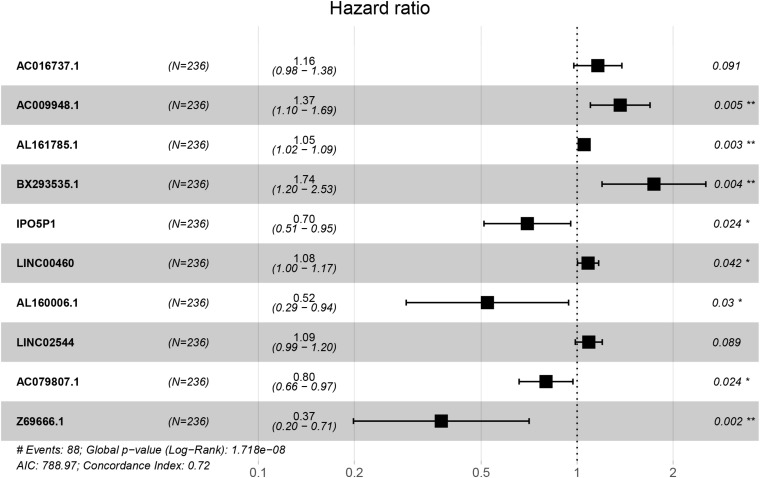
Forest plot of the prognostic value of the hypoxia-related lncRNAs.

**FIGURE 3:**
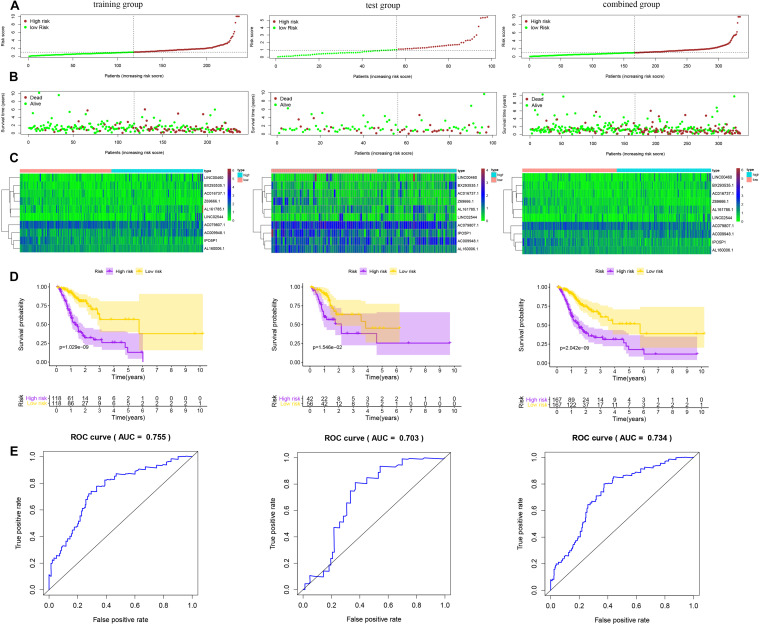
Risk score of the prognostic signature comprising ten hypoxia-related lncRNAs for overall survival (OS) in the three groups. **(A)** Distribution of patients with different risk scores in the training, test and combined groups. **(B)** OS status of patients with different risk scores in the training, test and combined groups. **(C)** Heatmap of the prognostic signature scores in the training, test and combined groups. **(D)** Kaplan–Meier (K-M) analysis of patients in the high- and low-risk groups in the training, test and combined groups. **(E)** Verification of the prognostic value of the hypoxia-related lncRNA signature by ROC analysis in the training, test and combined groups.

We used Cox regression analysis with the Lasso method in the training group to construct a hypoxia-related lncRNA signature for evaluating the prognosis of gastric cancer patients. Ultimately, 10 lncRNAs were selected to construct the model. The formula was as follows: Risk Score = [AC016737.1 × (0.14966)] + [AC009948.1 × (0.31130)] + [AL161785.1 × (0.05093)] + [BX293535.1 × (0.55486)] + [IPO5P1 × (-0.35954)] + [LINC00460 × (0.07925)] + [AL160006.1 × (-0.64798)] + [LINC02544 × (0.08429)] + [AC079807.1 × (-0.22459)] + [Z69666.1 × (-0.98107)] (Table 1 and Figure 2). Among the included lncRNAs, six (AL161785.1, LINC00460, LINC02544, AC016737.1, AC009948.1, and BX293535.1) were prognostic risk factors, and four (IPO5P1, AL160006.1, AC079807.1, and Z69666.1) were prognostic protective factors. The patients were divided into high- and low-risk groups according to the median risk score values calculated by the formula. The predictive power of the 10-lncRNA hypoxia-related prognostic signature for OS in patients is shown in Figure 3. Kaplan–Meier analysis with the log-rank test indicated that OS of patients in the high-risk group was lower than that of patients in the low-risk group (*P* < 0.05, Figure 3D). We then used ROC analysis to evaluate the prognostic accuracy of the model (Figure 3E) and observed an AUC value of 0.755 in the training group. In addition, we performed verification analysis of this 10-lncRNA signature in the test group and combined group. Survival analysis showed that the 5-year OS rate in the high-risk group was lower than that in the low-risk group (Figure 3D). The AUCs for the test group and combined group were 0.703 and 0.734, respectively (Figure 3E).

**TABLE 1:** Cox regression analysis with the Lasso method for the 10 hypoxia-related lncRNA prognostic signature (CI, confidence interval; HR, hazard ratio).

ID	Coef	HR	HR.95L	HR.95H	*P*-values
AC016737.1	0.14966	1.161	0.976	1.382	0.091
AC009948.1	0.31130	1.365	1.101	1.693	0.005
AL161785.1	0.05093	1.052	1.018	1.088	0.003
BX293535.1	0.55486	1.742	1.197	2.534	0.004
IPO5P1	-0.35954	0.698	0.510	0.955	0.024
LINC00460	0.07925	1.082	1.003	1.168	0.042
AL160006.1	-0.64798	0.523	0.291	0.940	0.030
LINC02544	0.08429	1.088	0.987	1.199	0.089
AC079807.1	-0.22459	0.799	0.658	0.970	0.024
Z69666.1	-0.98107	0.375	0.199	0.707	0.002

**FIGURE 4:**
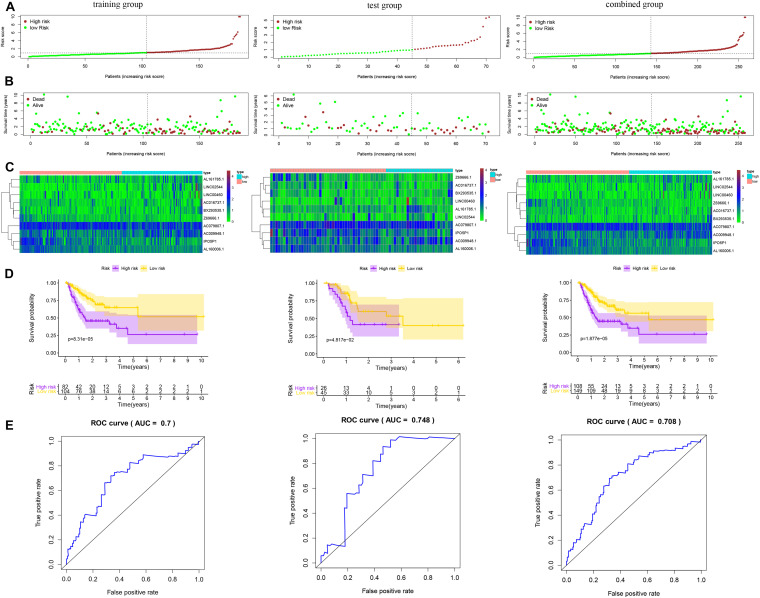
Risk score of the hypoxia-related lncRNA signature for disease-free survival (DFS) in the three groups. **(A)** Distribution of patients with different risk scores in the training, test and combined groups. **(B)** DFS of patients with different risk scores in the training, test and combined groups. **(C)** Heatmap of the prognostic signature scores in the training, test and combined groups. **(D)** K-M analysis of patients in the high- and low-risk groups in the training, test and combined groups. **(E)** Verification of the prognostic value of the hypoxia-related lncRNA signature by ROC analysis in the training, test and combined groups.

The predictive value of the 10-lncRNA hypoxia-related prognostic signature for DFS in patients by the same formula is displayed in Figure 4. Kaplan–Meier analysis showed that the 5-year DFS rate in the high-risk group was lower than that in the low-risk group among the training, test and combined groups (Figure 4D). Receiver operating characteristic analysis showed that the AUCs of DFS for these three groups were 0.700, 0.748, and 0.708, respectively (Figure 4E). These results show that the hypoxia-related lncRNA signature for OS and DFS can be used as a valuable prognostic indicator in gastric cancer.

### Subgroup Analysis and Cox Analysis of the Hypoxia-Related lncRNA Prognostic Signature

**FIGURE 5:**
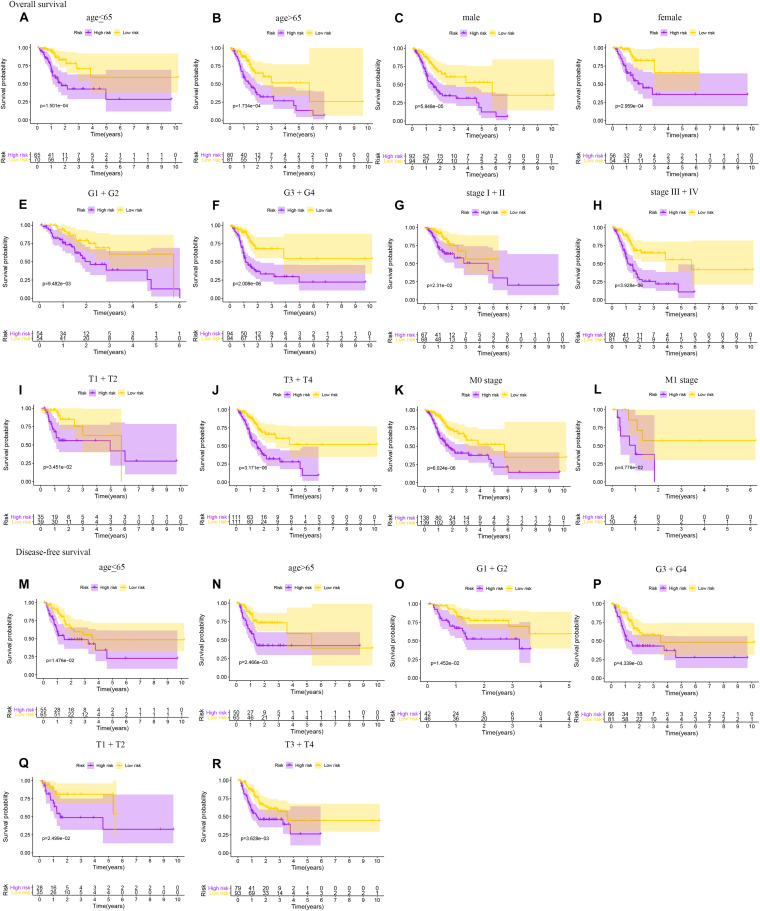
Subgroup analyses of OS and DFS for gastric cancer patients. OS: **(A)** age <65; **(B)** age > 65; **(C)** male; **(D)** female; **(E)** G1 + G2; **(F)** G3 + G4; **(G)** stage I + II; **(H)** stage III + IV; **(I)** T1 + T2; **(J)** T3 + T4; **(K)** M0 stage; **(L)** M1 stage. DFS: **(M)** age <65; **(N)** age > 65; **(O)** G1 + G2; **(P)** G3 + G4; **(Q)** T1 + T2; (R) T3 + T4.

**FIGURE 6:**
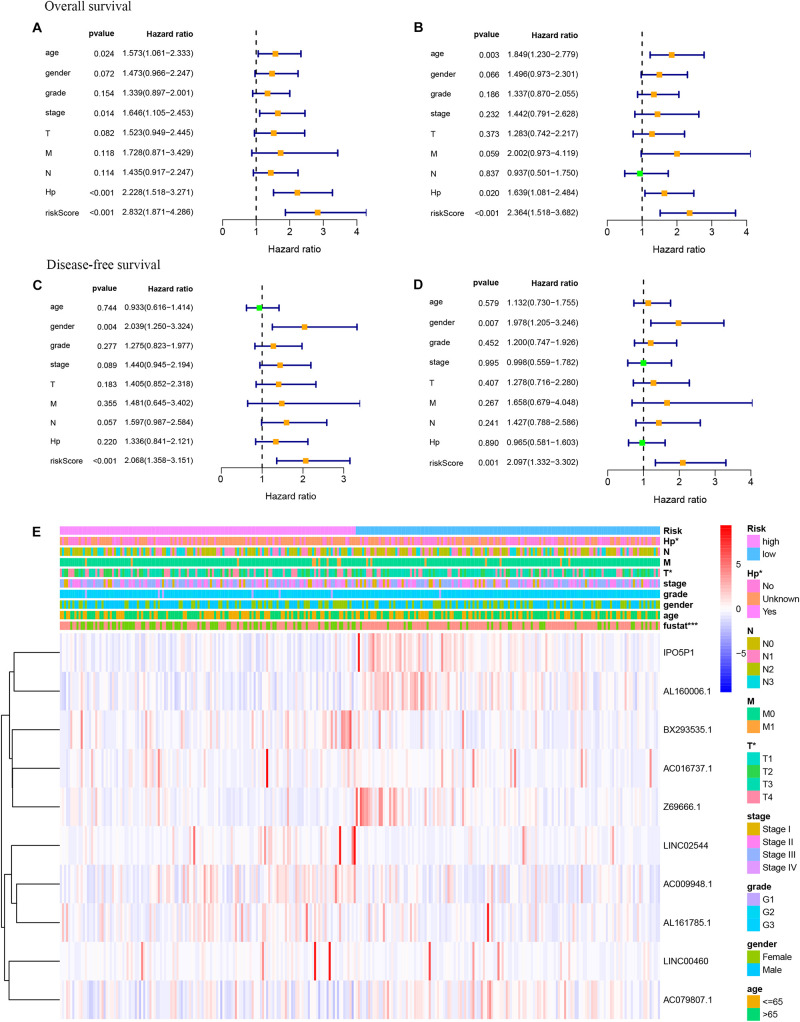
Univariate and multivariate Cox analyses of gastric cancer data. **(A)** Univariate analysis for OS prognosis. **(B)** Multivariate analysis for OS prognosis. **(C)** Univariate analysis for DFS prognosis. **(D)** Multivariate analysis for DFS prognosis. **(E)** Relationship between the risk score and clinical significance (***P < 0.001, **P < 0.01, *P < 0.05).

As shown in Figure 5, the hypoxia-related lncRNA prognostic signature can be used as a prognostic indicator for OS and DFS in subgroups of patients with different clinical characteristics. The results are shown for subgroups stratified by age (age <65 vs. age > 65), sex (male vs. female), grade (G1 + G2 vs. G3 + G4), clinical stage (stage I + II vs. stage III + IV), T stage (T1 + T2 vs. T3 + T4), and M stage (M0 vs. M1). In Figures 5A–L, the 5-year OS rates of the high-risk patients based on age, sex, grade, clinical stage, T stage, and M stage were lower than those of the low-risk patients. In Figures 5M–R, the 5-year DFS rates of the high-risk patients based on age, grade, and T stage were lower than those of the low-risk patients. To investigate whether the hypoxia-related lncRNA signature is an independent risk factor for the prognosis of OS and DFS in gastric cancer, univariate and multivariate Cox analyses were performed. Univariate Cox analysis showed that age (*P* = 0.024), stage (*P* = 0.014), Hp status (*P* < 0.001), and the hypoxia-related lncRNA signature (*P* < 0.001) were meaningful for predicting OS (Figure 6A). Multivariate Cox analysis showed that age (HR = 1.849, 95% CI = 1.230–2.779, *P* = 0.003), Hp (HR = 1.639, 95% CI = 1.081–2.484, *P* = 0.020), and the hypoxia-related lncRNA signature (HR = 2.364, 95% CI = 1.518–3.682, *P* < 0.001) were independent risk factors for predicting OS in gastric cancer (Figure 6B). In Figure 6C, univariate Cox analysis showed that gender (*P* = 0.004) and the hypoxia-related lncRNA signature (*P* < 0.001) were significant for predicting DFS. Multivariate Cox analysis showed that sex (HR = 1.978, 95% CI = 1.205–3.246, *P* = 0.007) and the hypoxia-related lncRNA signature (HR = 2.097, 95% CI = 1.332–3.302, *P* = 0.001) were independent risk factors for predicting DFS in gastric cancer (Figure 6D). In addition, using the chi-square test and Fisher’s exact probability test, we found that the risk score of the hypoxia-related lncRNA signature differed based on Hp status and T stage (Figure 6E).

### Construction and Evaluation of the Prognostic Nomogram

**FIGURE 7:**
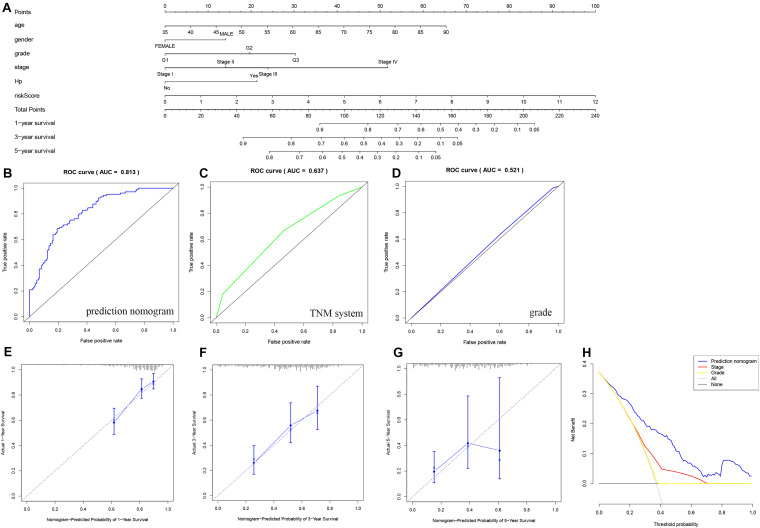
Nomogram used to predict the OS prognosis of patients with gastric cancer at 1, 3 and 5 years. **(A)** Nomogram based on the signature and clinical information. ROC analysis of OS prediction with the **(B)** nomogram, **(C)** TNM system, and **(D)** grade. **(E–G)** Calibration curve for the predictive accuracy of the nomogram. **(H)** Decision curve analysis evaluating the clinical practicality of the nomogram.

**FIGURE 8:**
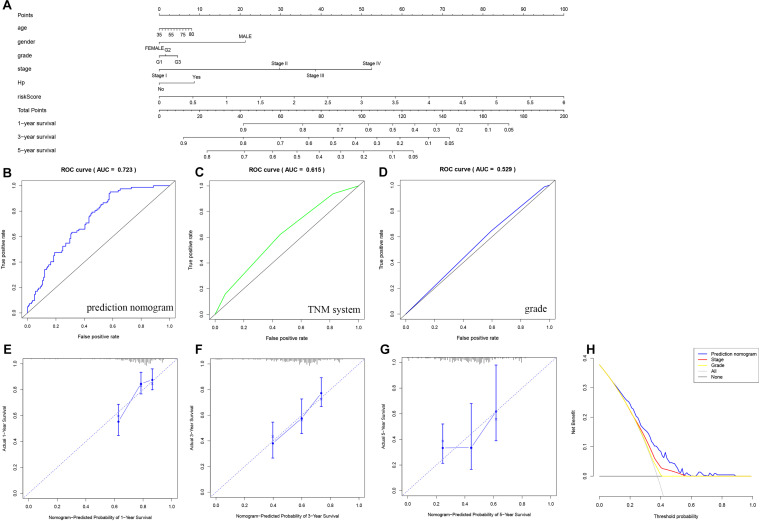
Nomogram used to predict the DFS prognosis of patients with gastric cancer at 1, 3 and 5 years. **(A)** Nomogram based on the signature and clinical information. ROC analysis of DFS prediction with the **(B)** nomogram, **(C)** TNM system, and **(D)** grade. **(E–G)** Calibration curve for the predictive accuracy of the nomogram. **(H)** Decision curve analysis evaluating the clinical practicality of the nomogram.

As shown in Figures 7A, 8A, we used the clinical characteristics and risk score to construct two new nomograms with the “rms” package in R software to predict the 1-, 3-, and 5-year OS and DFS rates of gastric cancer patients. Each factor (age, sex, grade, clinical stage, Hp status, and risk score) was used to obtain the corresponding score summary and the total score of the individual sample. The higher the sample score is, the worse the prognosis. The predicted AUC values of the OS and DFS nomograms were 0.813 and 0.723, respectively, which were higher than those of the TNM staging system (OS: AUC = 0.637, DFS: AUC = 0.615) and tumor grade (OS: AUC = 0.521, DFS: AUC = 0.529), indicating that the predictive power of the nomogram constructed here is more accurate than that of TNM status or tumor grade (Figures 7B–D, 8B–D). A calibration curve was used to indicate the consistency between the actual observed prognosis value and the value predicted by the nomogram. The calibration curves for 1-, 3-, and 5-year survival indicate a good fit for the nomogram (Figures 7E–G, 8E–G). As shown in Figures 7H, 8H, decision curve analysis was used to compare the net benefit to gastric cancer patients with different predictors. The abscissa shows the potential probability threshold; the ordinate, the net benefit. Compared with the TNM staging system and tumor grade, the predicted nomogram shows better clinical practicality for both OS and DFS.

### Biological Function Analysis of the Hypoxia-Related lncRNA Signature

**FIGURE 9:**
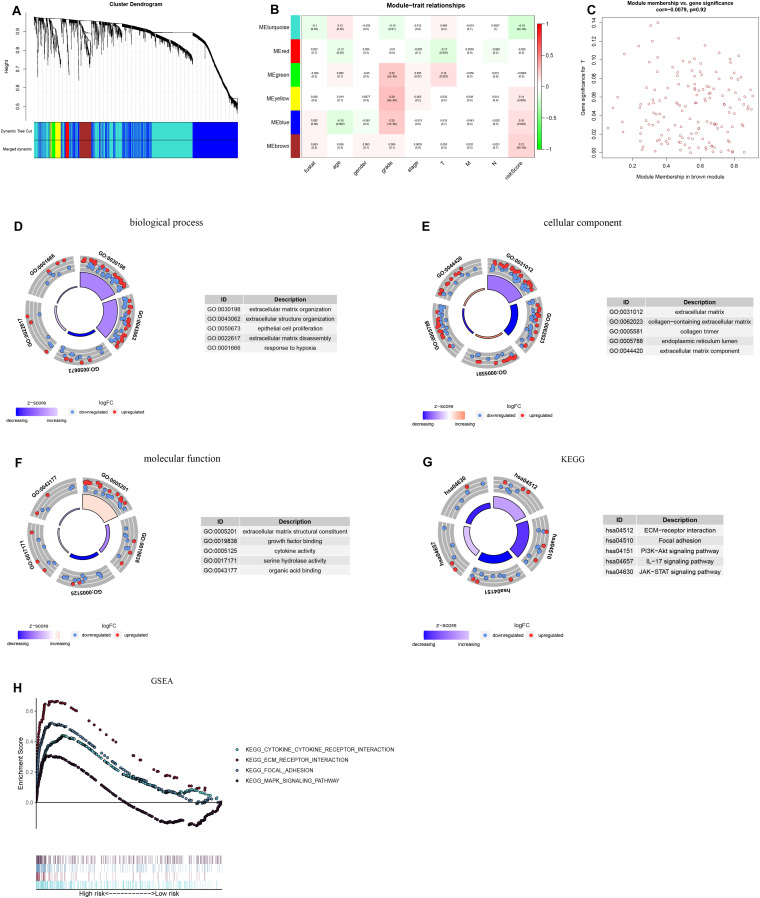
Weighted gene coexpression network analysis (WGCNA) and functional enrichment analysis. **(A)** Gene dendrogram and module color. **(B)** Correlations between gene modules and clinical information. **(C)** Associations of genes in the brown module with the risk score. GO analysis included the **(D)** biological process, **(E)** cellular component, and **(F)** molecular function. **(G)** KEGG enrichment analysis of target genes. **(H)** Enrichment plots from GSEA.

We constructed a lncRNA-mRNA coexpression network based on WGCNA to assess the biological function associated with the hypoxia-related lncRNA signature. The target genes were obtained from the set of differentially expressed mRNAs (| logFC| > 1, FDR < 0.05). A total of 6 modules were identified, among which the brown module was considered the most relevant to the risk score and contained 154 genes (Figures 9A–C). These genes were subjected to GO term and KEGG pathway enrichment analyses to elucidate their biological functions. The GO analysis comprises the biological process (BP), cellular component (CC), and molecular function (MF) categories. The BP category included the terms response to hypoxia and extracellular matrix organization (Figure 9D), the CC category was enriched mainly with the terms collagen trimer and extracellular matrix component (Figure 9E), and the MF category was enriched with the terms growth factor binding and cytokine activity (Figure 9F). KEGG pathway enrichment analysis showed that the genes were enriched in tumor-related signaling pathways, including the PI3K-Akt signaling pathway, IL-17 signaling pathway, and JAK-STAT signaling pathway, suggesting that activation of these pathways increases patients’ mortality risk (Figure 9G). In addition, GSEA was used to analyze the differences in pathway enrichment between the high- and low-risk groups (Figure 9H) and indicated enrichment in the pathways ECM-receptor interaction, focal adhesion, and cytokine-cytokine receptor interaction.

### Construction of a PPI Network and Verification of Target Genes

**FIGURE 10:**
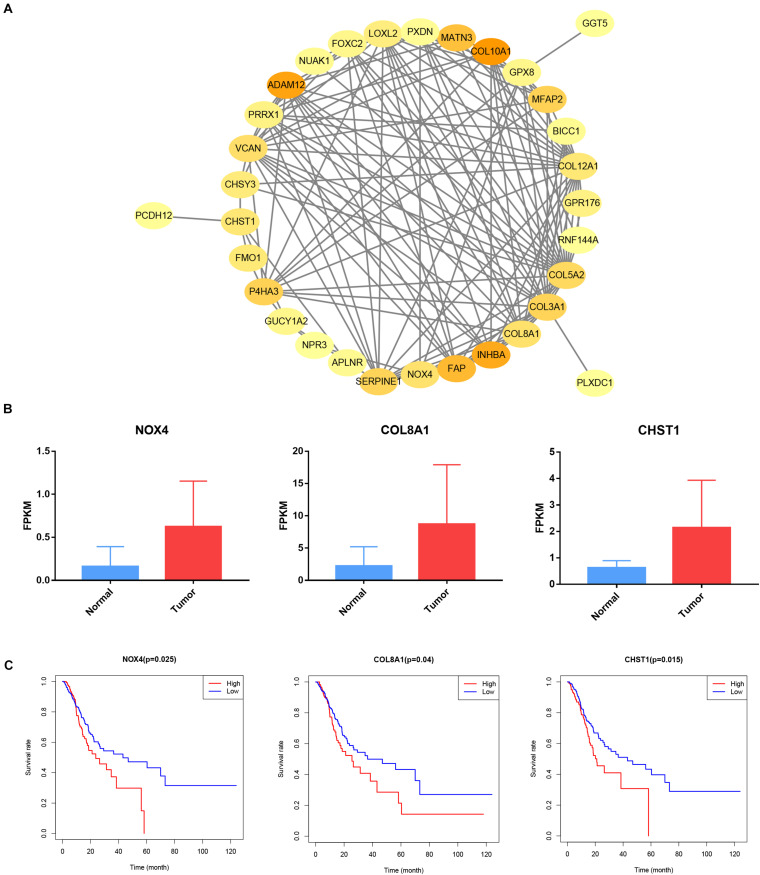
Construct protein-protein interaction networks and prognosis of target genes. **(A)** Cytoscape visualizes the genes of the interacting protein-protein interaction network. **(B)** NOX4, COL8A1 and CHST1 expression in STAD samples from TCGA database. **(C)** Prognostic associations of NOX4, COL8A1 and CHST1 from TCGA database.

**FIGURE 11:**
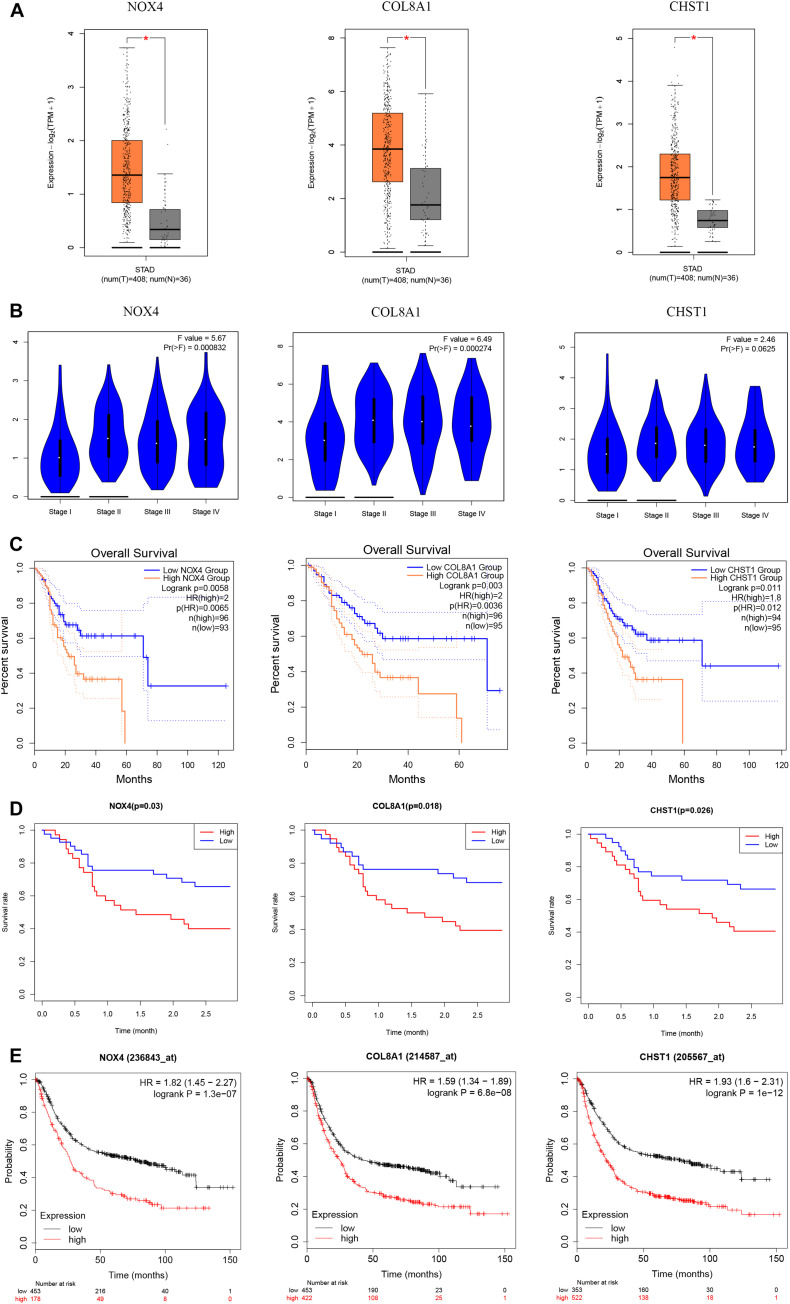
Expression and prognosis of NOX4, COL8A1, and CHST1 from the GEPIA, GEO and K-M Plotter databases. **(A)** Expression of these genes in gastric cancer tissues from the GEPIA database. **(B)** Expression of these genes based on tumor stage of tissues from the GEPIA database. **(C)** Prognostic associations of these genes with the cohort from the GEPIA database. **(D)** Prognostic associations of these genes with the cohort from the GEO database. **(E)** Prognostic associations of these genes with the cohort from the K-M Plotter database.

**FIGURE 12:**
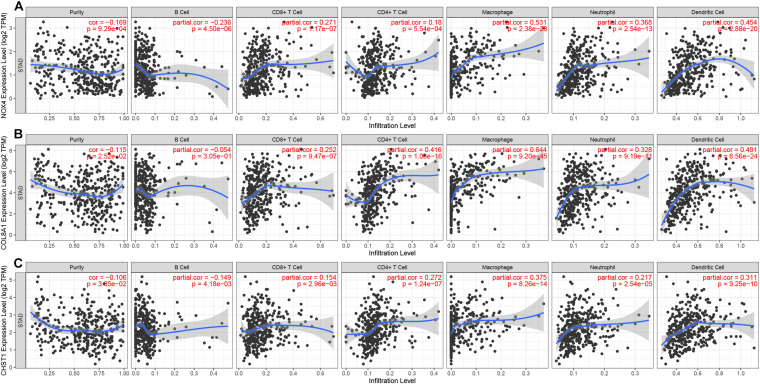
Correlation between gene expression and immune cell infiltration (TIMER). Correlation between the abundance of immune cells and the expression of **(A)** NOX4, **(B)** COL8A1, and **(C)** CHST1.

Our survival analysis of 154 genes in the brown module showed that 32 genes were related to the prognosis of gastric cancer. Then, we constructed a PPI network comprising 32 nodes and 138 edges via the STRING online database to reflect the interactions of target genes (Figure 10A). The interaction data of PPI network is summarized in Supplementary Table 1. After literature search, we selected 3 genes (NOX4, COL8A1, and CHST1) for further study. NOX4 (logFC = 1.948, *P* = 4.55e-12), COL8A1 (logFC = 1.982, *P* = 1.22e-8) and CHST1 (logFC = 1.783, *P* = 9.04e-13) were significantly upregulated in gastric cancer tissues (Figure 10B). In addition, the 5-year survival rates in the groups with high NOX4 (*P* = 0.025), COL8A1 (*P* = 0.040), and CHST1 (*P* = 0.015) expression were lower than those in the corresponding groups with low expression of these proteins (Figure 10C). We verified the expression levels and survival associations of these three genes in three external databases—GEPIA, GEO and K-M Plotter. In the cohort from the GEPIA database, the expression of NOX4, COL8A1, and CHST1 in gastric cancer tissues was higher than that in normal tissues, and the expression levels of NOX4 (*F* = 5.67 and *P* = 0.0008) and COL8A1 (*F* = 6.49 and *P* = 0.0003) differed across the four pathological stages (Figures 11A,B). As shown in Figure 11C, high mRNA expression levels were associated with poor prognosis (*P* < 0.05). In the GSE84426 dataset, the group with high expression of NOX4, COL8A1, and CHST1 had a shorter OS than did the low expression group (*P* < 0.05) (Figure 11D). In the 875 gastric cancer patients from the K-M Plotter database, the 5-year OS rate in the group with high expression of these 3 genes was lower than that in the group with low expression (*P* < 0.05) (Figure 11E). The analyses of gastric cancer cohorts from all three databases are consistent with those of our study. Meanwhile, by investigating a correlation between these three genes and immune cells in gastric cancer patients based on the TIMER website, we found that the expression of NOX4, COL8A1, and CHST1 was positively correlated with the degree of infiltration of CD8+ T cells, CD4+ T cells, macrophages, neutrophils, and dendritic cells in the immune microenvironment (*P* < 0.05) (Figure 12). The above data indicate that these lncRNA target genes may be involved in the formation of the tumor immune microenvironment.

## Discussion

Gastric cancer is the fifth most frequently diagnosed malignancy and the third-leading cause of tumor-related deaths worldwide (Bray et al., 2018). Despite the progress made in surgery, radiotherapy and chemotherapy, the prognosis of advanced gastric cancer is still poor. The TNM staging system is the current gold standard for evaluating tumor prognosis, but it does not consider genetic alterations, and patients with the same stage of disease often have different therapeutic and prognostic outcomes (Choi et al., 2017). Hypoxia is a microenvironmental feature of many tumors with poor prognosis and is a main cause of treatment failure. Therefore, identifying new prognostic markers is an ongoing challenge in biomedical research. Owing to the development of bioinformatics technology, researchers can screen for new prognostic markers in gastric cancer. Recently, lncRNA-based signatures have received increasing attention due to their improved predictive accuracy compared with that of standard benchmarks (Miao et al., 2019; Liu et al., 2020; Shen et al., 2020). However, prognostic markers based on hypoxia-related lncRNA expression profiles have not been studied in gastric cancer.

Our research focuses on hypoxia-related lncRNA signatures with prognostic value. In the training group, we initially identified 24 hypoxia-related lncRNAs associated with prognosis and constructed a prognostic signature comprising 10 lncRNA via Cox regression with the Lasso method. Kaplan–Meier analysis showed that both OS and DFS of patients with high risk scores were shorter than those of patients with low risk scores. In addition, the 10-lncRNA signature was confirmed by ROC curve analysis as a highly sensitive and specific prognostic marker in gastric cancer. In addition, the results were further verified in the test and combined groups. The 10-lncRNA signature was also associated with poor OS of gastric cancer patients in different subgroups, especially age, sex, tumor grade, clinical stage, T stage, and M stage, whereas the signature was associated with poor DFS of gastric cancer patients in only the age, tumor grade, and T stage subgroups. Furthermore, this signature was shown to be an independent risk factor for both OS and DFS. These results show that the hypoxia-related lncRNA signature can be a good indicator of gastric cancer prognosis compared with the predictive power of existing signatures reported in recent studies (Table 2; Cheng, 2018; Liu et al., 2018; Hu et al., 2019; Yu et al., 2019; Qi et al., 2020). Next, we constructed a nomogram to calculate a score representing both OS and DFS of gastric cancer. The calibration plot shows that the model has a satisfactory fit and better clinical practicality than the traditional TNM staging system. We constructed a lncRNA-mRNA coexpression network using WGCNA to analyze the role of the 10-lncRNA signature. The target genes in the brown module were enriched mainly in tumor-related pathways, such as the PI3K-Akt, IL-17 and JAK-STAT signaling pathways. In addition, the expression of the target genes NOX4, COL8A1 and CHST1 in the brown module was upregulated in gastric cancer, and these genes could predict the prognosis of gastric cancer patients, as verified in 3 independent cohorts. The expression of these three target genes is also positively correlated with the degree of immune cell infiltration in the gastric cancer tumor microenvironment, suggesting that NOX4, COL8A1 and CHST1 may be involved in the establishment of the tumor immune microenvironment.

**TABLE 2:** The comparison of studies about existing signatures for gastric cancer.

Databases	Signature	Symbols	Survival event	AUC value	References
TCGA	Six-mRNAs	TMEM132C, PCOLCE2, UPK1B, PM20D1, FLJ35024, and SLITRK2	OS	0.604	Hu et al., 2019
TCGA	Four-mRNAs	NDRG1, NDRG2, NDRG3, and NDRG4	OS	0.679	Yu et al., 2019
TCGA, GEO	Nine-mRNAs	CST2, AADAC, SERPINE1, COL8A1, SMPD3, ASPN, ITGBL1, MAP7D2, and PLEKHS1	OS	0.696	Liu et al., 2018
TCGA	Three-lncRNAs	CYP4A22−AS1, AP000695.6, and RP11-108M12.3	OS	0.660	Cheng, 2018
TCGA	Two-lncRNAs	LINC00106 and RP11-999E24.3	OS	0.614	Qi et al., 2020

Among the 10 key hypoxia-related lncRNAs found in gastric cancer, LINC00460 has been reported to be associated with poor prognosis in a variety of tumors. This lncRNA has been shown to be upregulated in gastric cancer and to be an independent risk factor for gastric cancer prognosis. Functional tests *in vivo* and *in vitro* have shown that LINC00460 can promote the proliferation of gastric cancer cells (Yang et al., 2020). Chaudhary et al. reported that high expression of LINC00460 in head and neck squamous cell carcinoma is associated with poor prognosis (Chaudhary et al., 2020). Another study showed that colorectal cancer patients with high expression of LINC00460 have a higher incidence of tumor metastasis and that this lncRNA is an independent risk factor for prognosis (Zhang et al., 2019). In EGFR-mutant lung cancer, LINC00460 promotes EGFR-TKI resistance, and patients with high LINC00460 expression who receive gefitinib therapy have a lower five-year OS rate than do patients with low LINC00460 expression (Nakano et al., 2020). Lian et al. discovered that elevated expression of LINC00460 in osteosarcoma is often accompanied by distant metastasis and poor prognosis, and this lncRNA is expected to become a therapeutic target in osteosarcoma (Lian et al., 2019). However, the nine other lncRNAs were reported for the first time in gastric cancer and are worthy of future research. Regarding the functions and target genes regulated by lncRNAs in the hypoxia-related prognostic signature, the literature states that activation of the PI3K-Akt signaling pathway plays a key role in gastric cancer (Singh et al., 2015). Cai et al. revealed that ANTXR1 promotes the proliferation, invasion and migration of gastric cancer cells through the PI3K-Akt signaling pathway and inhibits apoptosis, which is related to poor prognosis (Cai et al., 2020). The JAK-STAT signaling pathway affects tumorigenesis and development and is a therapeutic target in cancer (Pencik et al., 2016; Groner and von Manstein, 2017). In non-small cell lung cancer, the lncRNA PART1 has been reported to affect the tumorigenic ability of lung cancer cells *in vivo* through the JAK-STAT signaling pathway (Zhu et al., 2019). The target gene NOX4 plays a key role in the development of gastric cancer. Du et al. (2019) revealed that NOX4 is highly expressed in gastric cancer tissues and that high expression of NOX4 is associated with poor prognosis in gastric cancer. NOX4 can promote the resistance of gastric cancer cells to anoikis by upregulating ROS and EGFR, resulting in distant metastasis (Du et al., 2018). Another target gene, COL8A1, has been reported to affect tumor progression. Using WGCNA, Shang et al. (2018) found that COL8A1 is a core gene in colon adenocarcinoma and is highly expressed in tumor tissues. Indeed, it is an independent risk factor for colon adenocarcinoma prognosis. In liver cancer, silencing the expression of COL8A1 inhibits the proliferation and invasion of tumor cells (Zhao et al., 2009). The above reports are consistent with our research. However, the relationships between the 10-lncRNA signature and its target genes and pathways are still unclear. To provide new directions for the specific treatment of gastric cancer, more in-depth research on the molecular mechanisms of the 10 lncRNAs included in the signature must be conducted.

Nomograms are widely used in the evaluation of tumor prognosis (Iasonos et al., 2008; Balachandran et al., 2015). The degree to which the various factors in the model contribute to the outcome is scored, and the predicted value of individual outcome events is calculated by determining the total score of the different factors. The main advantage of a nomogram is that it can make individualized risk assessments according to the characteristics of patients or diseases. Many nomograms have been used in individualized prognosis prediction for different cancers, such as renal cell carcinoma, prostate cancer, cervical cancer, and lung cancer (Halabi et al., 2014; Liang et al., 2015; Rose et al., 2015; Wei et al., 2019). In our study, we constructed a prognostic nomogram combining clinical features with a hypoxia-related lncRNA signature. More importantly, the accuracy and clinical value of our nomogram for predicting prognosis were higher than those of the traditional TNM staging system.

Although the hypoxia-related lncRNA signature was determined to be stable in our research, our study has some limitations. First, our research population mainly comprised patients and samples from international databases and were internally verified. We still need to confirm the accuracy of the hypoxia-related lncRNA signature in other databases and on local data as external validation. Second, some hypoxia-related lncRNAs have rarely been reported in the academic literature, PPI, and the mechanisms of action of hypoxia-related lncRNAs in gastric cancer need to be elucidated with *in vivo* and *in vitro* experiments.

In summary, we constructed a prognostic signature comprising 10 hypoxia-related lncRNAs to predict both OS and DFS of gastric cancer and verified this signature in different data sets. Furthermore, this predictive nomogram model based on our established signature of 10 hypoxia-related lncRNAs provides better clinical value than the traditional TNM staging system for predicting the prognosis of gastric cancer patients. We anticipate that this signature will provide a new reference for current predictions of gastric cancer prognosis and offer new ideas for individualized treatment.

## Data Availability Statement

Publicly available datasets were analyzed in this study. This data can be found here: https://cancergenome.nih.gov/; https://www.ncbi.nlm.nih.gov/geo/.

## Author Contributions

QC conceived and designed the experiments and wrote the manuscript. All authors analyzed the data, reviewed, and approved the final manuscript.

## Conflict of Interest

The authors declare that the research was conducted in the absence of any commercial or financial relationships that could be construed as a potential conflict of interest.

## Footnotes

1 https://cancergenome.nih.gov/

2 http://www.ensembl.org/index.html

3 https://www.ncbi.nlm.nih.gov/geo/

4 https://www.gsea-msigdb.org/gsea/msigdb/

5 http://http://gepia.cancer-pku.cn

6 http://kmplot.com/analysis/

7 https://cistrome.shinyapps.io/timer/

## References

[B1] Balachandran V. P. Gonen M. Smith J. J. DeMatteo R. P. (2015). Nomograms in oncology: more than meets the eye. *Lancet Oncol.* 16 173–e180. 10.1016/S1470-2045(14)71116-7 PMC4465353 25846097

[B2] Bhan A. Soleimani M. Mandal S. S. (2017). Long noncoding RNA and cancer: a new paradigm. *Cancer Res.* 77 3965–3981. 10.1158/0008-5472.CAN-16-2634 28701486 PMC8330958

[B3] Bray F. Ferlay J. Soerjomataram I. Siegel R. L. Torre L. A. Jemal A. (2018). Global cancer statistics 2018: GLOBOCAN estimates of incidence and mortality worldwide for 36 cancers in 185 countries. *CA Cancer J. Clin.* 68 394–424. 10.3322/caac.21492 30207593

[B4] Cai C. Dang W. Liu S. Huang L. Li Y. Li G. (2020). Anthrax toxin receptor 1/tumor endothelial marker 8 promotes gastric cancer progression through activation of the PI3K/AKT/mTOR signaling pathway. *Cancer Sci.* 111 1132–1145. 10.1111/cas.14326 31977138 PMC7156833

[B5] Chaudhary R. Wang X. Cao B. De La Iglesia J. Masannat J. Song F. (2020). Long noncoding RNA. LINC00460, as a prognostic biomarker in head and neck squamous cell carcinoma (HNSCC). *Am. J. Transl. Res.* 12 684–696. 32194915 PMC7061833

[B6] Cheng P. (2018). A prognostic 3-long noncoding RNA signature for patients with gastric cancer. *J. Cell. Biochem.* 119 9261–9269. 10.1002/jcb.27195 30074647

[B7] Choi K. H. Kim B. S. Oh S. T. Yook J. H. Kim B. S. (2017). Comparison the sixth and seventh editions of the AJCC staging system for T1 gastric cancer: a long-term follow-up study of 2124 patients. *Gastr. Cancer* 20 43–48. 10.1007/s10120-015-0590-0 26732877

[B8] Du S. Miao J. Lu X. Shi L. Sun J. Xu E. (2019). NADPH oxidase 4 is correlated with gastric cancer progression and predicts a poor prognosis. *Am. J. Transl. Res.* 11 3518–3530. 31312363 PMC6614607

[B9] Du S. Miao J. Zhu Z. Xu E. Shi L. Ai S. (2018). NADPH oxidase 4 regulates anoikis resistance of gastric cancer cells through the generation of reactive oxygen species and the induction of EGFR. *Cell Death Dis.* 9:948. 10.1038/s41419-018-0953-7 30237423 PMC6148243

[B10] Groner B. von Manstein V. (2017). Jak Stat signaling and cancer: opportunities, benefits and side effects of targeted inhibition. *Mol. Cell Endocrinol.* 451 1–14. 10.1016/j.mce.2017.05.033 28576744

[B11] Halabi S. Lin C. Y. Kelly W. K. Fizazi K. S. Moul J. W. Kaplan E. B. (2014). Updated prognostic model for predicting overall survival in first-line chemotherapy for patients with metastatic castration-resistant prostate cancer. *J. Clin. Oncol.* 32 671–677. 10.1200/JCO.2013.52.3696 24449231 PMC3927736

[B12] Harris A. L. (2002). Hypoxia–a key regulatory factor in tumour growth. *Nat. Rev. Cancer* 2 38–47. 10.1038/nrc704 11902584

[B13] Hu B. Xie M. Li K. Li J. Gui Y. Xu J. (2019). Genome-wide analysis to identify a novel distant metastasis-related gene signature predicting survival in patients with gastric cancer. *Biomed. Pharmacother.* 117:109159. 10.1016/j.biopha.2019.109159 31247467

[B14] Huang L. Lin H. Kang L. Huang P. Huang J. Cai J. (2019). Aberrant expression of long noncoding RNA SNHG15 correlates with liver metastasis and poor survival in colorectal cancer. *J. Cell Physiol.* 234 7032–7039. 10.1002/jcp.27456 30317592

[B15] Iasonos A. Schrag D. Raj G. V. Panageas K. S. (2008). How to build and interpret a nomogram for cancer prognosis. *J. Clin. Oncol.* 26 1364–1370. 10.1200/JCO.2007.12.9791 18323559

[B16] Li H. Wang X. Wen C. Huo Z. Wang W. Zhan Q. (2017). Long noncoding RNA NORAD, a novel competing endogenous RNA, enhances the hypoxia-induced epithelial-mesenchymal transition to promote metastasis in pancreatic cancer. *Mol. Cancer* 16:169. 10.1186/s12943-017-0738-0 29121972 PMC5679488

[B17] Lian H. Xie P. Yin N. Zhang J. Zhang X. Li J. (2019). Linc00460 promotes osteosarcoma progression via miR-1224-5p/FADS1 axis. *Life Sci.* 233:116757. 10.1016/j.lfs.2019.116757 31419446

[B18] Liang W. Zhang L. Jiang G. Wang Q. Liu L. Liu D. (2015). Development and validation of a nomogram for predicting survival in patients with resected non-small-cell lung cancer. *J. Clin. Oncol.* 33 861–869. 10.1200/JCO.2014.56.6661 25624438

[B19] Liu X. Wu J. Zhang D. Bing Z. Tian J. Ni M. (2018). Identification of Potential Key Genes Associated With the Pathogenesis and Prognosis of Gastric Cancer Based on Integrated Bioinformatics Analysis. *Front. Genet.* 9:265. 10.3389/fgene.2018.00265 30065754 PMC6056647

[B20] Liu Y. Wang L. Liu H. Li C. He J. (2020). The Prognostic Significance of Metabolic Syndrome and a Related Six-lncRNA Signature in Esophageal Squamous Cell Carcinoma. *Front. Oncol.* 10:61. 10.3389/fonc.2020.00061 32133283 PMC7040247

[B21] Lu X. Kang Y. (2010). Hypoxia and hypoxia-inducible factors: master regulators of metastasis. *Clin. Cancer Res.* 16 5928–5935. 10.1158/1078-0432.CCR-10-1360 20962028 PMC3005023

[B22] Martens-Uzunova E. S. Bottcher R. Croce C. M. Jenster G. Visakorpi T. Calin G. A. (2014). Long noncoding RNA in prostate, bladder, and kidney cancer. *Eur. Urol.* 65 1140–1151. 10.1016/j.eururo.2013.12.003 24373479

[B23] Miao R. Ge C. Zhang X. He Y. Ma X. Xiang X. (2019). Combined eight-long noncoding RNA signature: a new risk score predicting prognosis in elderly non-small cell lung cancer patients. *Aging* 11 467–479. 10.18632/aging.101752 30659574 PMC6366982

[B24] Nakano Y. Isobe K. Kobayashi H. Kaburaki K. Isshiki T. Sakamoto S. (2020). Clinical importance of long noncoding RNA LINC00460 expression in EGFRmutant lung adenocarcinoma. *Int. J. Oncol.* 56 243–257. 10.3892/ijo.2019.4919 31789388 PMC6910175

[B25] Pencik J. Pham H. T. Schmoellerl J. Javaheri T. Schlederer M. Culig Z. (2016). JAK-STAT signaling in cancer: from cytokines to non-coding genome. *Cytokine* 87 26–36. 10.1016/j.cyto.2016.06.017 27349799 PMC6059362

[B26] Peng W. Jiang A. (2016). Long noncoding RNA CCDC26 as a potential predictor biomarker contributes to tumorigenesis in pancreatic cancer. *Biomed. Pharmacother.* 83 712–717. 10.1016/j.biopha.2016.06.059 27470572

[B27] Ponting C. P. Oliver P. L. Reik W. (2009). Evolution and functions of long noncoding RNAs. *Cell* 136 629–641. 10.1016/j.cell.2009.02.006 19239885

[B28] Qi M. Yu B. Yu H. Li F. (2020). Integrated analysis of a ceRNA network reveals potential prognostic lncRNAs in gastric cancer. *Cancer Med.* 9 1798–1817. 10.1002/cam4.2760 31923354 PMC7050084

[B29] Rose P. G. Java J. Whitney C. W. Stehman F. B. Lanciano R. Thomas G. M. (2015). Nomograms Predicting Progression-Free Survival. Overall Survival, and Pelvic Recurrence in Locally Advanced Cervical Cancer Developed From an Analysis of Identifiable Prognostic Factors in Patients From NRG Oncology/Gynecologic Oncology Group Randomized Trials of Chemoradiotherapy. *J. Clin. Oncol.* 33 2136–2142. 10.1200/JCO.2014.57.7122 25732170 PMC4477785

[B30] Shang J. Wang F. Chen P. Wang X. Ding F. Liu S. (2018). Co-expression Network Analysis Identified COL8A1 Is Associated with the Progression and Prognosis in Human Colon Adenocarcinoma. *Dig. Dis. Sci.* 63 1219–1228. 10.1007/s10620-018-4996-5 29497907

[B31] Shen Y. Peng X. Shen C. (2020). Identification and validation of immune-related lncRNA prognostic signature for breast cancer. *Genomics* 112 2640–2646. 10.1016/j.ygeno.2020.02.015 32087243

[B32] Singh S. S. Yap W. N. Arfuso F. Kar S. Wang C. Cai W. (2015). Targeting the PI3K/Akt signaling pathway in gastric carcinoma: a reality for personalized medicine? *World J. Gastroenterol.* 21 12261–12273. 10.3748/wjg.v21.i43.12261 26604635 PMC4649111

[B33] Wang Z. Qin B. (2019). Prognostic and clinicopathological significance of long noncoding RNA CTD-2510F5.4 in gastric cancer. *Gastr. Cancer* 22 692–704. 10.1007/s10120-018-00911-x 30560474 PMC6570689

[B34] Wei J. H. Feng Z. H. Cao Y. Zhao H. W. Chen Z. H. Liao B. (2019). Predictive value of single-nucleotide polymorphism signature for recurrence in localised renal cell carcinoma: a retrospective analysis and multicentre validation study. *Lancet Oncol.* 20 591–600. 10.1016/S1470-2045(18)30932-X 30880070

[B35] Xie Y. Zhang Y. Du L. Jiang X. Yan S. Duan W. (2018). Circulating long noncoding RNA act as potential novel biomarkers for diagnosis and prognosis of non-small cell lung cancer. *Mol. Oncol.* 12 648–658. 10.1002/1878-0261.12188 29504701 PMC5928376

[B36] Yang J. Lian Y. Yang R. Lian Y. Wu J. Liu J. (2020). Upregulation of lncRNA LINC00460 Facilitates GC Progression through Epigenetically Silencing CCNG2 by EZH2/LSD1 and Indicates Poor Outcomes. *Mol. Ther. Nucl. Acids* 19 1164–1175. 10.1016/j.omtn.2019.12.041 32059342 PMC7016164

[B37] Yu C. Hao X. Zhang S. Hu W. Li J. Sun J. (2019). NDRG Characterization of the prognostic values of the family in gastric cancer. *Therap. Adv. Gastroenterol.* 12:1756284819858507. 10.1177/1756284819858507 31384305 PMC6647212

[B38] Zhang H. Lu Y. Wu J. Feng J. (2019). LINC00460 Hypomethylation Promotes Metastasis in Colorectal Carcinoma. *Front. Genet.* 10:880. 10.3389/fgene.2019.00880 31632435 PMC6779110

[B39] Zhao X. Liu Y. Yu S. (2017). Long noncoding RNA AWPPH promotes hepatocellular carcinoma progression through YBX1 and serves as a prognostic biomarker. *Biochim. Biophys. Acta Mol. Basis Dis.* 1863 1805–1816. 10.1016/j.bbadis.2017.04.014 28428004

[B40] Zhao Y. Jia L. Mao X. Xu H. Wang B. Liu Y. (2009). siRNA-targeted COL8A1 inhibits proliferation, reduces invasion and enhances sensitivity to D-limonence treatment in hepatocarcinoma cells. *IUBMB Life* 61 74–79. 10.1002/iub.151 19109829

[B41] Zhu D. Yu Y. Wang W. Wu K. Liu D. Yang Y. (2019). Long noncoding RNA PART1 promotes progression of non-small cell lung cancer cells via JAK-STAT signaling pathway. *Cancer Med.* 8 6064–6081. 10.1002/cam4.2494 31436388 PMC6792487

[B42] Zhu G. Wang S. Chen J. Wang Z. Liang X. Wang X. (2017). Long noncoding RNA HAS2-AS1 mediates hypoxia-induced invasiveness of oral squamous cell carcinoma. *Mol. Carcinog.* 56 2210–2222. 10.1002/mc.22674 28485478

